# 

**Published:** 1887-05

**Authors:** 


### TABLE OF CONTENTS.

### Original and Selected Articles.

Notes of Professor Gaston's Lecture on Malignant Tumors, by J. E Kintz, M. D., of Somerset, Ohio .......... 177
Cases of Scarlet Fever, by H. Christopher, M. D., of St. Joseph, Mo .......... 182
Veratrum Viride in the Treatment of Acute Pneumonia, by James B. Baird, M. D., Atlanta, Ga .......... 184
Supplementary Report of Ligation of External Iliac for Aneurism of Femoral Artery, by J. McF. Gaston, M.D., Atlanta, Ga .......... 186
The Treatment of Pruritus Pudendi, by E. S. McKee, M.D., of Cincinnati, Ohio .......... 187
A Case .......... 190
The "Elephant Man," Illustrated .......... 191
Pulse Invariability in Menstruation, Regardless of Posture; The Teeth from a Medico-legal Aspect .......... 193

### Abstracts and Gleanings.

The Treatment of Pneumonia .......... 194
Diphtheria; Treatment of Chorea .......... 195
Arseniate of Strychnine .......... 196
A New Method of Dilating the Uterus .......... 197
The Constipation Habit; The Management of Incomplete Abortion .......... 198
Overdose of Cocaine .......... 199
Salt Baths in Fevers; Nitro-Glycerin .......... 200
Action of Caffein and Theine; Antifebrine; Acute Rheumatism in Philadelphia Hospitals .......... 201
How Long to Wait Before Applying the Forceps .......... 202
Antifebrine; Transmission of Symptoms of Hysteria Under the Influence of a Magnet. 203
Typhoid from a Single Dose; Antiseptic Treatment of Croup .......... 204
Buttermilk in Sick Stomach; Coniin Hydrobromate; Canabin Tannate .......... 205
A Cure for Warts; Arsenite of Strychnine; Adonidin; Antifebrine; Scoparian and Spartein; To Remove Stains of Iodine from the Skin .......... 206

### Scientific Items.

Proving the Soundness of an Eye; Hei Schiller; Professional Fatigue .......... 207
Vegetable Meat-Eaters; A Pocket Corkscrew. 208

### Practical Notes and Formulæ.

Speedy Cure for Gonorrhœa; The Treatment of Threadworms in Children .......... 209
A Disinfectant Mixture for Apartments; Coughs; For Rheumatism .......... 210
Cimicifugia for Headache; Instantaneous Remedy for Lumbago; Atropine in Nocturnal Earaches of Children; Itch Ointment; Incontinence of Urine; Cotton Root as a Uterine Hæmostatic .......... 211

### Editorials and Miscellaneous.

Editorial Brevities; Antipyrine .......... 212
Qinine Not Good in Hemorrhagic Malarial Fever; Professor Jones' Medical and Surgical Memoirs; In Memery of J. E. Kintz .......... 213
Medical Association of Georgia—1887 .......... 214
Who is He; Books and Pamphlets Received .......... 217
Hydramnion .......... 219
Recepts; Special Notices .......... 220

